# Dual targeting heterodimer PET tracer [^18^F]AlF‑FAPI‑RGD in patients with rheumatoid arthritis: a pilot exploratory study

**DOI:** 10.7150/thno.102627

**Published:** 2024-10-21

**Authors:** Hao Wang, Wei Su, Limeng He, Dongze Wu, Nan Liu, Jing Zhu, Jingjing Zhang, Wei Zhang

**Affiliations:** 1 Department of Nuclear Medicine, Sichuan Provincial People's Hospital, University of Electronic Science and Technology of China, Chengdu, Sichuan, China.; 2 Department of Rheumatology and Immunology, Sichuan Provincial People's Hospital, University of Electronic Science and Technology of China, Chengdu, Sichuan, China.; 3 Department of Diagnostic Radiology, Yong Loo Lin School of Medicine, National University of Singapore, Singapore 119074, Singapore.; 4 Clinical Imaging Research Centre, Centre for Translational Medicine, Yong Loo Lin School of Medicine, National University of Singapore, Singapore 117599, Singapore.; 5 Theranostics Center of Excellence, Yong Loo Lin School of Medicine, National University of Singapore, 11 Biopolis Way, Helios, Singapore 138667, Singapore.; 6 Nanomedicine Translational Research Program, NUS Center for Nanomedicine, Yong Loo Lin School of Medicine, National University of Singapore, Singapore 117597, Singapore.

**Keywords:** rheumatoid arthritis, fibroblast activation protein, integrin, neoangiogenesis, [^18^F]AlF‑FAPI‑RGD PET/CT

## Abstract

**Purpose:** Fibroblast-like synoviocytes and angiogenesis play crucial roles in the advancement of rheumatoid arthritis (RA). This prospective study aimed to assess the efficacy of [^18^F]AlF-FAPI-RGD, a dual-targeting heterodimer tracer that focuses on fibroblast activation protein (FAP) and integrin α_v_β_3_, through PET/CT imaging for evaluating disease activity and response to treatment in RA.

**Methods:** Twenty-eight participants with active RA (12 males and 16 females; mean age, 55 ± 9 years) underwent clinical evaluation of disease activity and [^18^F]AlF-FAPI-RGD PET/CT imaging at enrollment. Subsequently, after a 3-month period, a follow-up scan and clinical assessments were conducted on these participants. Imaging parameters such as PET-positive joint count (PJC), PET-positive articular index (PAI), average SUV_max_ (aSUV_max_), and highest SUV_max_ (hSUV_max_) in affected joints were compared with clinical and laboratory findings, as well as traditional imaging modalities.

**Results:** [^18^F]AlF-FAPI-RGD PET/CT imaging produced high-quality images, revealing notable tracer uptake in the synovium of affected joints. [^18^F]AlF-FAPI-RGD demonstrated a higher positivity rate in detecting affected joints compared to the tender or swollen joint counts during clinical assessment (82.4% [342 of 415] vs 68.4% [284 of 415], respectively). Additionally, this imaging method successfully identified lung lesions with atypical respiratory symptoms in participants with RA. Following treatment, PJC, PAI, aSUV_max_, and hSUV_max_ values significantly decreased in responders (*P* < 0.001), while no significant changes were observed in non-responders (*P* > 0.05). Furthermore, a notable association was found between the percentage change in certain PET parameters and modifications in specific clinical parameters.

**Conclusion:** [^18^F]AlF-FAPI-RGD PET/CT represents a promising tool for the objective assessment of disease activity and treatment response in patients with RA. Furthermore, it may offer a novel imaging method for the early detection of subclinical RA and interstitial lung disease present with atypical respiratory symptoms.

## Introduction

Rheumatoid arthritis (RA), recognized as one of the most common autoimmune diseases, is marked by polyarticular synovitis which can lead to joint destruction throughout the body 1. An accurate assessment of disease activity is essential to formulating an effective treatment strategy for RA. The disease activity score with 28-joint counts (DAS28) is a commonly utilized clinical tool that evaluates disease activity by considering factors such as swollen joints, tender joints, the acute phase response, and overall health 2. Nevertheless, DAS28 might involve subjectivity 3 and lacks the sensitivity needed for early or subclinical disease detection 4. Synovial tissue is the main site of RA activity 5. In comparison to traditional imaging methods such as X-rays, ultrasound, and magnetic resonance imaging (MRI), molecular imaging technology may offer enhanced sensitivity and specificity in visualizing lesions within the synovial tissue. Recent studies have explored the use of ^18^F-fluoro-2-deoxyglucose (^18^F-FDG) positron emission tomography/computed tomography (PET/CT) in assessing RA disease activity. These studies have shown that this imaging technique enables the sensitive detection of active RA lesions and is also valuable for monitoring therapeutic outcomes 6-10. However, it is crucial to understand that ^18^F-FDG PET/CT, while promising, serves as a non-specific marker of inflammation and may show elevated uptake in several inflammatory conditions other than RA 11.

During RA progression, the synovial membrane experiences hyperplasia and invasion, resulting in the deterioration of both cartilage and bone. Fibroblast-like cells (FLSs) that coat the joint surface play a pivotal role in these destructive mechanisms 12. Fibroblast activation protein (FAP), an enzyme located on cell surfaces and part of the serine protease dipeptidyl peptidase family, has abnormal expression patterns not only in cancerous conditions but also in inflammatory and nonmalignant fibrotic tissues 13-15. Ge and colleagues introduced [^18^F]AlF-NOTA-FAPI-04 as a tool for visualizing RA-FLSs both in laboratory settings and in inflamed joints of collagen-induced arthritis mice and RA patients. This highlighted the tracer's specificity to inflamed joints 16. A recent study by Luo et al. 17 explored the utility of FAP-targeted PET/CT imaging in RA subjects. Using ^68^Ga-FAPI-04, they identified notably increased FAPI absorption in inflamed joints, with the number of affected joints on the PET/CT scan aligning with the patient's disease activity. Subsequently, Pan et al. 18 determined that ^68^Ga-FAPI-04 PET/CT scans could potentially forecast treatment responses in RA patients. Angiogenesis stands out as a crucial feature of synovial tissue in RA starting from the early stages of the disease, playing a key role in the development of proliferative synovium 19. The integrin α_v_β_3_, which is highly expressed on new blood vessels, interacts with radiotracers that include the Arg-Gly-Asp (RGD) sequence. Several preclinical and clinical studies have shown selective accumulation of RGD in the synovium of joints affected by RA 20, 21. A preliminary study by Zhu et al. suggested that ^68^Ga-PRGD_2_ PET/CT is effective in evaluating synovial angiogenesis and monitoring treatment responses in RA patients 22. In a more extensive cohort study by Kavanal et al., findings indicated that ^68^Ga-RGD2 PET/CT imaging serves as an objective method for assessing disease activity and treatment response in RA patients 23.

The aforementioned studies have validated the significance of FAP and neoangiogenesis in synovitis, endorsing the targeted clinical imaging of these factors in RA patients. Nevertheless, challenges in the application of single-target receptors may emerge due to disease heterogeneity, resulting in inadequate tracer uptake, leading to missed lesions. Prior research has indicated that the ^68^Ga-labeled FAPI-RGD heterodimer surpassed its monomeric counterparts in a mouse model with tumors 24. This radiotracer has demonstrated clinical utility for tumors expressing integrin and FAP in patients, producing superior image quality 25, 26. The [^18^F]AlF-FAPI-RGD, comparable in performance to [^68^Ga] Ga-FAPI-RGD, generated high-quality images in both animal studies and initial human trials in our preliminary investigation 27. Further evaluation of [^18^F]AlF-FAPI-RGD as a novel PET tracer is necessary to assess its effectiveness in assessing RA joint disease activity.

## Materials and Methods

### Study design and participants

This prospective exploratory study was conducted at Sichuan Provincial People's Hospital. Approval was obtained from the institutional review board (No. 2023-287) and the study was carried out in compliance with the 1964 Declaration of Helsinki and its subsequent revisions, or equivalent ethical standards. The study was also registered at ClinicalTrials.gov (Identifier #NCT05944198).

In our study, we recruited a total of 28 participants with RA between July 2023 and February 2024. Each participant provided written informed consent. Inclusion criteria for participants in this study required a clinical diagnosis of definite RA based on the 2010 American College of Rheumatology/European League Against Rheumatism (ELAR) classification criteria 28, being 18 years of age or older, and being able to comprehend and sign an informed consent form. At the time of enrollment, the disease activity of RA was assessed clinically by two experienced rheumatologists. Participants exhibiting moderate to high disease activity, as determined by the clinical disease activity index, were included in the study. Exclusion criteria involved participants with known malignancy or other autoimmune diseases, those who were pregnant or lactating, and individuals with claustrophobia or other contraindications to PET/CT scanning. All participants underwent plain radiography of both hands and wrists, as well as [^18^F]AlF-FAPI-RGD PET/CT, conducted within one week before initiating or adjusting rheumatoid arthritis medication. Joint destruction was evaluated using the van der Heijde modified total Sharp score (mTSS) using radiography of both the hands 29. Higher mTSS scores indicate a greater degree of joint destruction. Following the imaging procedures, all participants received treatment based on the recommendations of the rheumatologists, who were unaware of the PET/CT results. Participants were clinically monitored for a duration of three months.

### Clinical assessment of disease activity and response criteria

The participants underwent a thorough clinical assessment that included a physical exam, blood work, and patient evaluation. During the physical exam, the focus was on evaluating the joints, specifically examining the tender joint count (TJC) and swollen joint count (SJC) using a 28-joint assessment (shoulder, elbow, wrist, metacarpophalangeal joint, proximal interphalangeal joint, knee). Blood samples were taken at the beginning to measure rheumatoid factor (RF), anti-citrullinated protein antibodies (ACPA), erythrocyte sedimentation rate (ESR) and C-reactive protein (CRP). Patient evaluation included assessing pain, patient global evaluation (PGA), and evaluator global evaluation (EGA) of disease activity using visual analogue scales (VAS). Disease activity in RA was determined by calculating the clinical disease activity index (CDAI), simplified disease activity index (SDAI), and DAS28 with either ESR or CRP 30. Response criteria were defined based on the percentage reduction in CDAI or SDAI from baseline: significant response (≥ 85% reduction), moderate response (≥ 70% reduction), minor response (≥ 50% reduction), and no response (< 50% reduction) 31.

### Synthesis of radiopharmaceuticals

[^18^F]AlF‑FAPI‑RGD was radiolabeled using an automated method 27 and the labelling time was approximately 45 min. The apparent molar activity of [^18^F]AlF‑FAPI‑RGD was 20-120 MBq/nmol, with 36.6 ± 2.4% radiolabeling yield and > 99% radiochemical purity according to radio-HPLC analysis. Additionally, [^18^F]AlF‑FAPI‑RGD remained stable in saline and serum for up to 2 h.

### PET/CT imaging acquisition

No particular preparations were conducted for participants before undergoing PET/CT scans. The administered dose of [^18^F]AlF-FAPI-RGD varied between 3.70 and 4.81 MBq (0.10-0.13 mCi) per kilogram of body weight. Following an average uptake time of 80.3 ± 11.2 min post-injection, PET/CT imaging was executed from the top of the skull down to the toes, with the arms positioned downward. This procedure utilized a specialized PET/CT scanner (Biograph mCT Flow 64, Siemens, Germany) that incorporates FlowMotion scanning technology and CT attenuation correction. The CT parameters included settings of 120 keV, 50 mAs, a pitch of 1.3, slice thickness of 3 mm, and a rotation time of 0.5 s. Furthermore, regional PET/CT images covering the bilateral hands and wrist joints area were obtained using a low-dose CT at 40 mA. The collected data were then reconstructed employing the ordered subset expectation maximization method, utilizing 2 iterations and 21 subsets.

### Imaging analysis

The two experienced nuclear medicine physicians independently analyzed the PET/CT images, each with over 5 years of experience in PET/CT imaging, in a randomized fashion. Clinical information was blinded to readers, who then achieved a consensus on interpreting the images. Any discrepancies in opinion were resolved through this consensus process. Joints exhibiting greater tracer uptake compared to the background were identified as positive, while those with uptake equal to or less than the background were labeled as negative. To evaluate FAPI-RGD uptake in 28 joints, regions of interest were delineated for each joint and the maximum standardized uptake value (SUV_max_) was calculated. Spherical regions of interest, with a 1-cm diameter, were positioned in the ascending aorta (representing the blood pool) and the gluteus maximus (representing muscle) for background assessment. The locations and intensity of joints were documented on a three-point scale relative to blood pool and muscle uptake levels 18. The scoring system was defined with the following criteria: joints received 0 points if there was no uptake observed; 1 point if the uptake in joints was equal to or higher than muscle uptake but not exceeding the blood pool uptake; 2 points if the joint uptake was greater than the blood pool uptake but did not exceed twice the blood pool uptake; and 3 points if the joint uptake was more than twice the blood pool uptake. Then, calculations were made for each participant for the PET joint count (PJC, which is the number of joints showing RA activity on PET), the PET articular index (PAI, representing the sum of scores for each joint on a scale of three points), the average SUV_max_ of PET-positive joints (aSUV_max_), and the highest SUV_max_ observed among PET-positive joints (hSUV_max_) 17, 18.

### Statistical analysis

The study utilized SPSS 22.0 (SPSS Inc., Chicago, IL) for statistical analyses. A normality assessment was performed on the quantitative variable. Continuous variables were expressed as mean ± SD or median with appropriate range. Intergroup comparisons were made using the Student's *t*-test for data with normal distribution and Wilcoxon test for skewed data (Shapiro-Wilk test for normality). Comparison of initial and follow-up PET parameters was performed using a paired *t* test for data with normal distribution and paired samples Wilcoxon test for skewed data (Shapiro-Wilk test for normality). Categorical variables were represented as rates or percentages and analyzed with the Chi-square test. Correlation studies applied Pearson correlation coefficients for normally distributed data and Spearman rank correlation coefficients for skewed data. For correction of multiple comparisons, Bonferroni method was performed to control the rates of false-positive findings. Statistical significance was set at a two-tailed *P*-value of <0.05.

## Results

### Clinical characteristics of the study population

The study included twenty-eight participants (twelve men and 16 women; 55 years ± 9) having either new-onset active disease or a history of RA with relapsing or continuous active disease determined by DAS28-ESR. The median disease duration was 56.5 months (range 1-370 months). More detailed characteristics of the study population can be found in Table 1 and Supplementary Table S1.

### Detection of affected joints and other lesions with [^18^F]AlF-FAPI-RGD PET/CT

**Figure 1:**
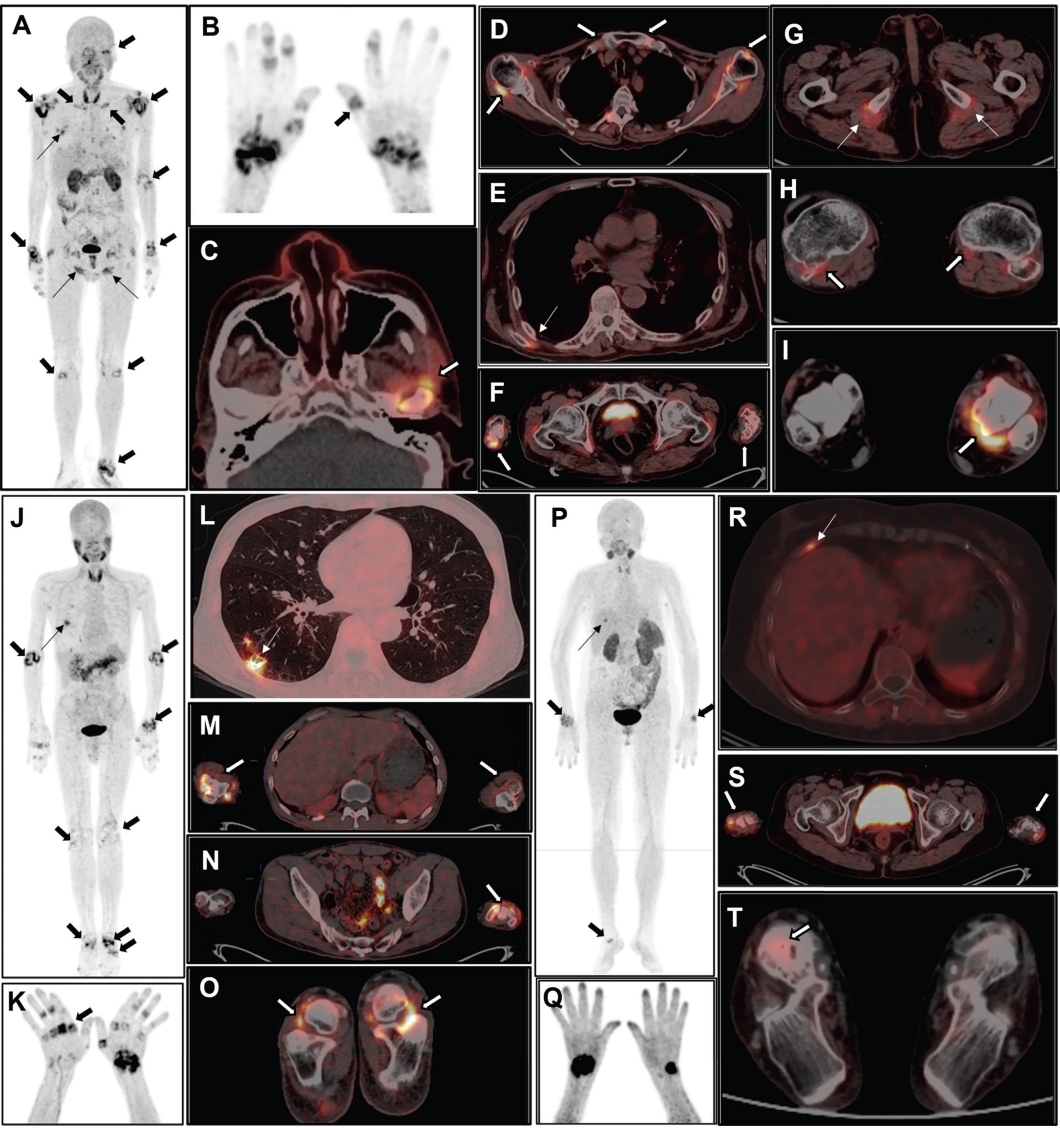
The maximum intensity projection (MIP) images (A and B) of a 60-year-old male participant (No. 7) reveal increased uptake in various joints (thick arrows), muscles and tissues (thin arrows). The participant has DAS28-ESR score of 5.6, DAS28-CRP score of 5.2, CDAI of 16, and SDAI of 219. Axial PET/CT fusion images demonstrate elevated FAPI-RGD uptake in the left temporomandibular joint (C), bilateral shoulder and sternoclavicular joints (D), bilateral paraspinal muscles of the subscapularis angle (E), bilateral carpal joints (F), bilateral tissues around the sciatic tuberosity (G), bilateral knee joints (H), and the left ankle joint (I). The MIP images (J and K) of a 53-year-old male participant (No. 14) reveal increased uptake in multiple joints (thick arrows) and right lung (thin arrow). The participant has DAS28-ESR score of 8.5, DAS28-CRP score of 7.2, CDAI of 69.5, and SDAI of 75.9. Axial PET/CT fusion images reveal increased FAPI-RGD uptake in the lower lobe of the right lung (L), bilateral elbow joints (M), left wrist joint (N), and bilateral ankle joints (O). The MIP images (P and Q) of a 54-year-old female participant (No. 5) show increased uptake in the joints (thick arrow) and right lower thorax (thin arrow). The participant has DAS28-ESR score of 3.3, DAS28-CRP score of 2.3, CDAI of 6.0, and SDAI of 6.5. Axial PET/CT fusion images reveal increased FAPI-RGD uptake in the region of the right rib fracture (R), bilateral wrist joints (S), and right ankle joint (T).

**Figure 2:**
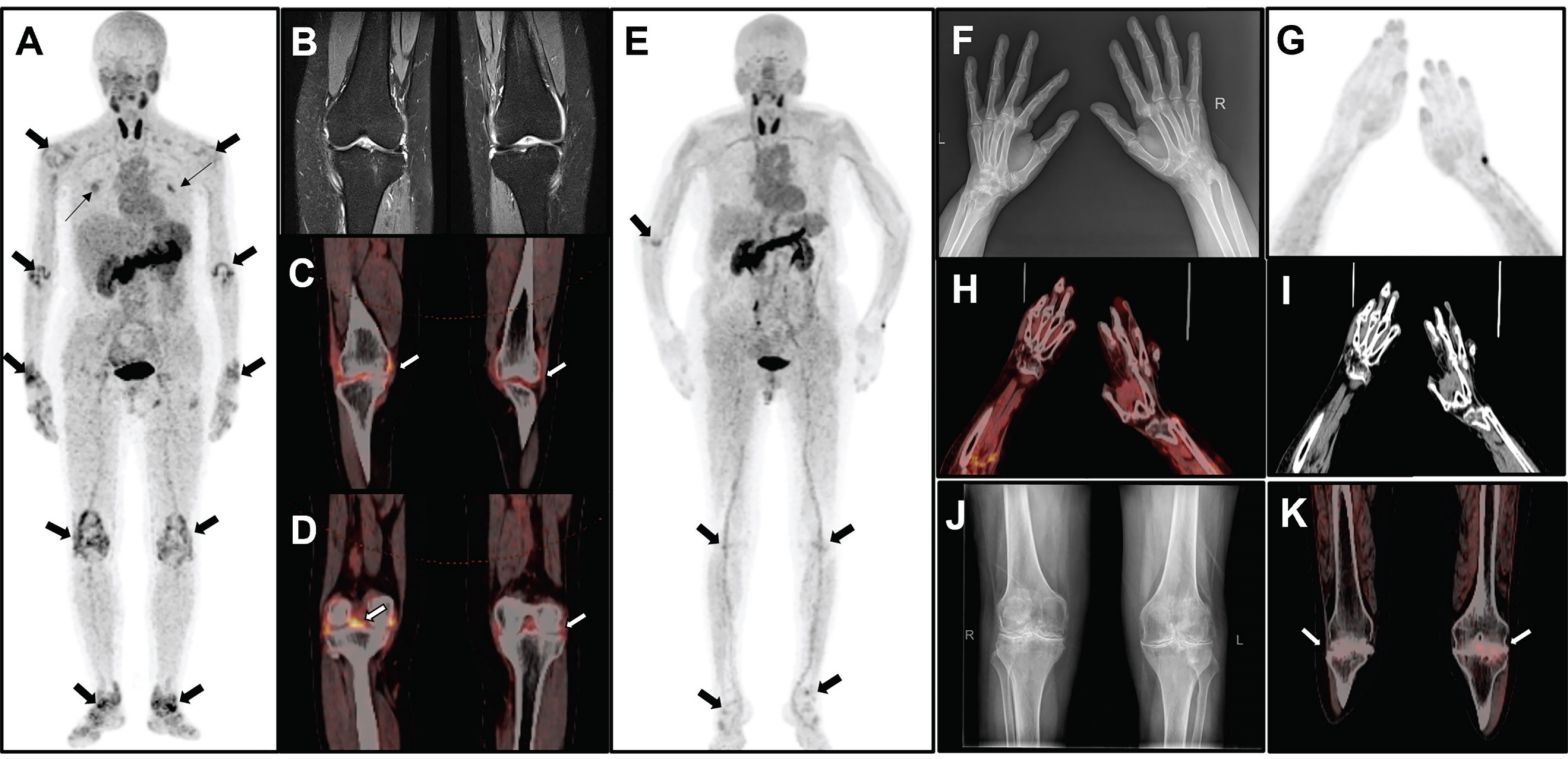
The MIP image (A) obtained from a 51-year-old female participant (No. 2) displays increased uptake in multiple joints (thick arrows) and muscles (thin arrows). The participant has DAS28-ESR score of 6.9, DAS28-CRP score of 5.9, CDAI of 38.0, and SDAI of 39.4. Bilateral knee MRI T2 images (B) reveals thickening of the knee capsule, suprapatellar bursa, and popliteal synovium. Axial PET/CT fusion images (C and D) demonstrate increased uptake of FAPI-RGD in these specific areas. The MIP image (E) of a 69-year-old female participant (No. 18) reveals mild to moderate increased uptake in various joints (thick arrows). The participant has DAS28-ESR score of 4.4, DAS28-CRP score of 3.8, CDAI of 10.0, and SDAI of 32.1. X-ray imaging (F) of both hands and wrists reveals slightly narrowed and blurred interphalangeal joint spaces, soft tissue swelling around the joints, cystic degeneration in both hands and wrists, and an unclear wrist joint space. MIP image (G) alongside PET/CT images (H, fusion; I, CT) of the hand and wrist region reveals osteoporosis and some deformities, with no significant uptake at the corresponding sites. Bilateral knee radiograph (J) shows bilateral tibial plateaus, femoral epicondyles and patellar rim osteophytes, with significant narrowing of the joint space, local fusion, and uneven bone density under the articular surfaces. The axial PET/CT fusion image (K) shows a bilateral narrowing of the knee joint space and an increased mild FAPI-RGD uptake.

Physiological locations where [^18^F]AlF-FAPI-RGD is taken up were observed in various parts of the body, such as the thyroid gland, liver, spleen, pancreas, kidneys, and urinary bladder. Furthermore, there was varying uptake in the salivary glands, nail beds, intestinal loops, and bone marrow. A consistently low distribution of [^18^F]AlF-FAPI-RGD in other areas allowed for an accurate evaluation of joint inflammation in study participants. Pathologically elevated tracer uptake was observed in the synovium of the joints. Representative PET/CT images can be seen in Figure 1 and Figure 2.

**Figure 3:**
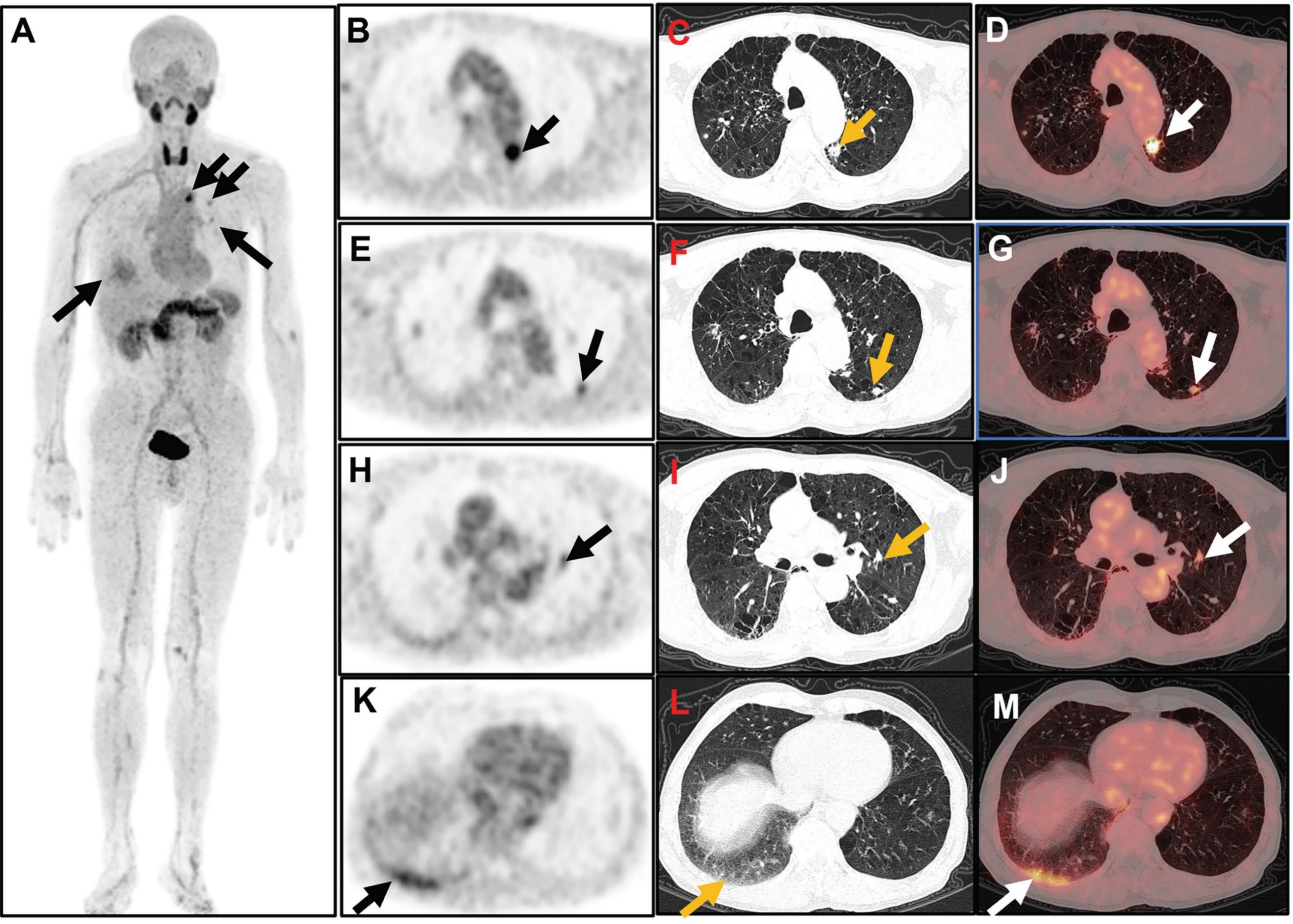
[^18^F]AlF-FAPI-RGD-PET images of a 59-year-old man (Participant 17) with a 38-month history of RA and ongoing disease activity. The participant has a DAS28-ESR score of 6.1, DAS28-CRP score of 5.5, CDAI of 31.0, and SDAI of 45.2. Whole-body MIP image (A) shows various degrees of increased uptake of FAPI-RGD at multiple sites in the bilateral chest. Axial PET/CT images revealed increased FAPI-RGD uptake in a small patch in the upper left lung (arrow; B, PET; C, CT; D, fusion; SUV_max_ 8.1), a small nodule in the dorsal segment of the lower lobe of the left lung (arrow; E, PET; F, CT; G, fusion; SUV_max_ 3.6), a small nodule near the left hilar (arrow; H, PET; I, CT; J, fusion; SUV_max_ 3.3), and a ground-glass density patchy shadow at the base of the right lung (arrow; K, PET; L, CT; M, fusion; SUV_max_ 4.3).

All 28 participants showed positive results for arthritis detection using [^18^F]AlF-FAPI-RGD PET/CT. Out of the 784 joints in the 28 joint regions evaluated, PET/CT identified 342 positive joints. Apart from the 28 joint regions, an extra 145 positive joints were found, including various areas like the temporomandibular joint (n = 5), distal interphalangeal joint (n = 12), atlantoaxial joint (n = 7), hip joint (n = 4), sternoclavicular joint (n = 10), ankle joint (n = 31), metatarsophalangeal joint (n = 31), and interphalangeal joint of the lower extremities (n = 45). Lung lesions were present in 22 out of 28 participants, with manifestations such as nodules, plaques, streaks, and ground-glass opacities showing varying levels of FAPI-RGD uptake (Figure 3). Furthermore, FAPI-RGD uptake was seen in the paraspinal muscles of the subscapularis angle in 5 participants, in the tissues surrounding the sciatic tuberosity in 6 participants, and in one participant, a high FAPI-RGD uptake focus was detected in the region of a rib fracture.

Among these participants, PJC, PAI, and hSUV_max_ did not show significant differences between RA patients with new-onset active disease and those with relapsing or continuous active disease. However, the aSUV_max_ in RA patients with new-onset active disease was higher than that observed in patients with longstanding active RA (4.3 ± 0.6 vs 3.2 ± 1.2, *P* < 0.05) (Supplementary Table S2). Additional results of [^18^F]AlF-FAPI-RGD PET/CT in other positive joints and lesions in non-articular tissues of all 28 recruited participants are available in Supplementary Table S3.

### Correlation of PET/CT images with clinical parameters

The cohort of 28 participants had a mean DAS28-ESR of 6.4 ± 1.5 and DAS28-CRP of 5.4 ± 1.3. Among the 784 joints examined using 28-joint counts, 342 joints showed increased uptake of FAPI-RGD, in comparison to 284 joints identified as tender or swollen through clinical assessment. By combining physical examination and PET/CT, a total of 415 affected joints were identified. Of these, 211 joints displayed positive findings on both physical examination and [^18^F]AlF-FAPI-RGD PET/CT, while 131 joints did not exhibit tenderness or swelling but had positive findings on PET/CT. Furthermore, 73 tender or swollen joints did not show [^18^F]AlF-FAPI-RGD uptake. The positive predictive value for detecting affected joints was 68.4% (284 out of 415) for physical examination and 82.4% (342 out of 415) for [^18^F]AlF-FAPI-RGD PET/CT. The rate of RA joints detected by both physical examination and [^18^F]AlF-FAPI-RGD PET/CT was 50.8% (211 out of 415).

Table 2 displays the quantitative PET/CT variables (PJC, PAI, aSUV_max_, and hSUV_max_) obtained from [^18^F]AlF-FAPI-RGD PET/CT scans, along with the results of clinical disease activity assessments. Among the initial participant-specific PET parameters, PJC exhibited positive correlations with ESR, TJC, DAS28-ESR, DAS28-CRP, and CDAI. Similarly, PAI demonstrated positive correlations with TJC, DAS28-ESR, DAS28-CRP, and CDAI. Furthermore, hSUV_max_ showed positive correlations with ESR, DAS28-CRP, and SDAI, whereas no significant correlation was found between aSUV_max_ and the results of clinical disease activity assessments. Notably, there was no significant correlation observed between any of the PET parameters and van der Heijde mTSS (*P* > 0.05).

### Follow-up PET/CT after treatment

**Figure 4:**
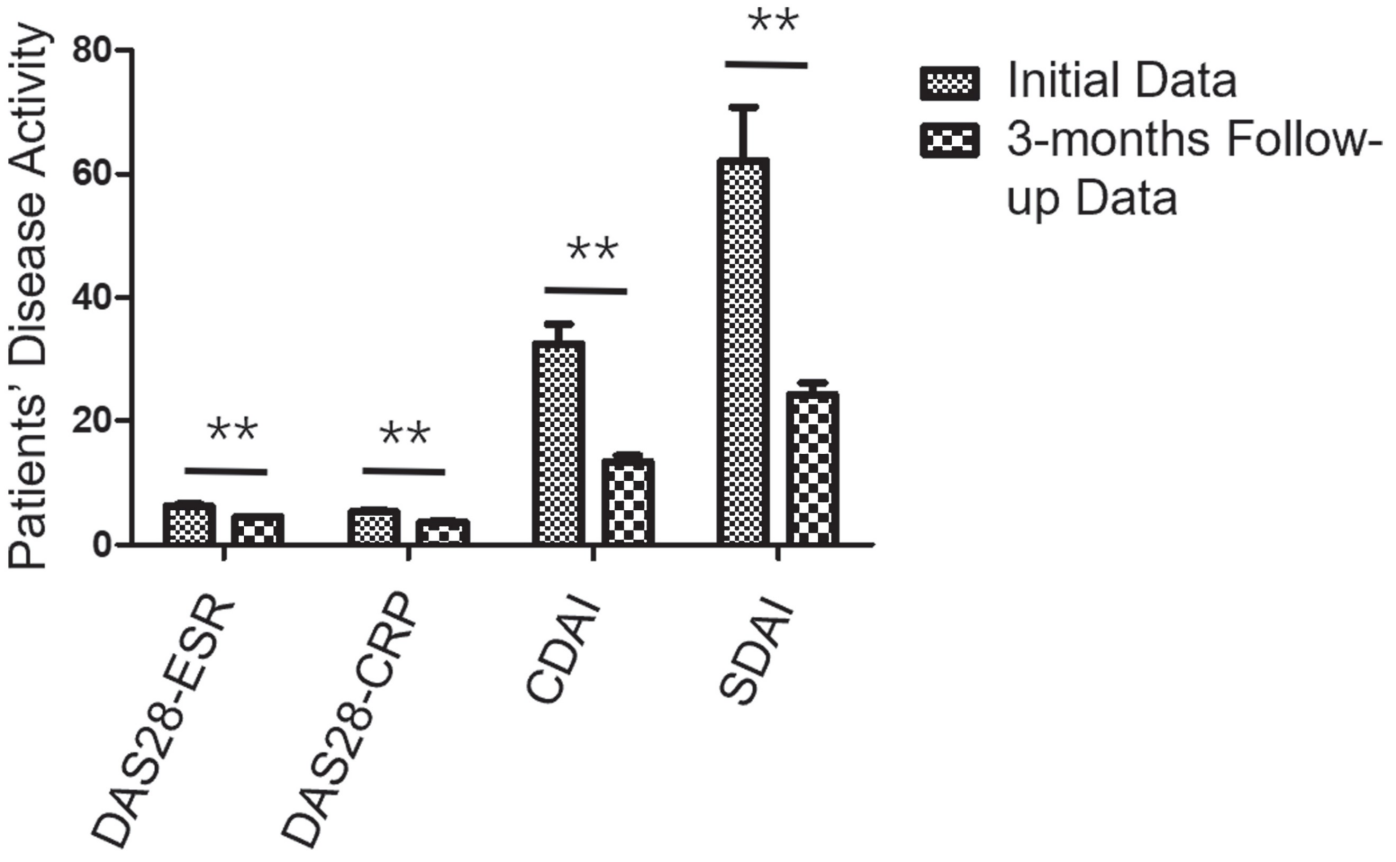
The changes in disease activity among RA participants undergoing treatment. Over a 3-month follow-up period, all 28 participants exhibited notable reductions in DAS28-ESR, DAS28-CRP, CDAI, and SDAI compared to their pre-treatment levels, with scores decreasing from 6.4 ± 1.5, 5.4 ± 1.3, 34.0 ± 16.5, and 65.6 ± 47.9 to 4.5 ± 0.5, 3.7 ± 0.5, 13.4 ± 5.2, and 24.2 ± 10.6, respectively (all *P* < 0.001). ^**^ *P* < 0.01.

Following baseline [^18^F]AlF-FAPI-RGD PET/CT imaging, all 28 participants were treated with conventional disease-modifying antirheumatic drugs (DMARDs) (including methotrexate, leflunomide, and hydroxychloroquine), with 5 participants also receiving regimens containing biological synthetic DMARDs (including tocilizumab and adalimumab). Additionally, eighteen participants were given prednisone and 11 received non-steroidal anti-inflammatory drugs. After a 3-month follow-up, DAS28-ESR, DAS28-CRP, CDAI, and SDAI showed a median percentage decline of 18.9%, 24.5%, 43.7%, and 40.0% respectively. Based on CDAI response, 20 participants achieved response (major response: 0 participant, moderate response: 8 participants, minor response: 12 participants), while 8 participants did not respond. According to SDAI response, 20 participants were responders (major response: 1 participant, moderate response: 8 participants, minor response: 11 participants), and 8 participants were non-responders. Changes in disease activity during treatment are illustrated in Figure 4.

**Figure 5:**
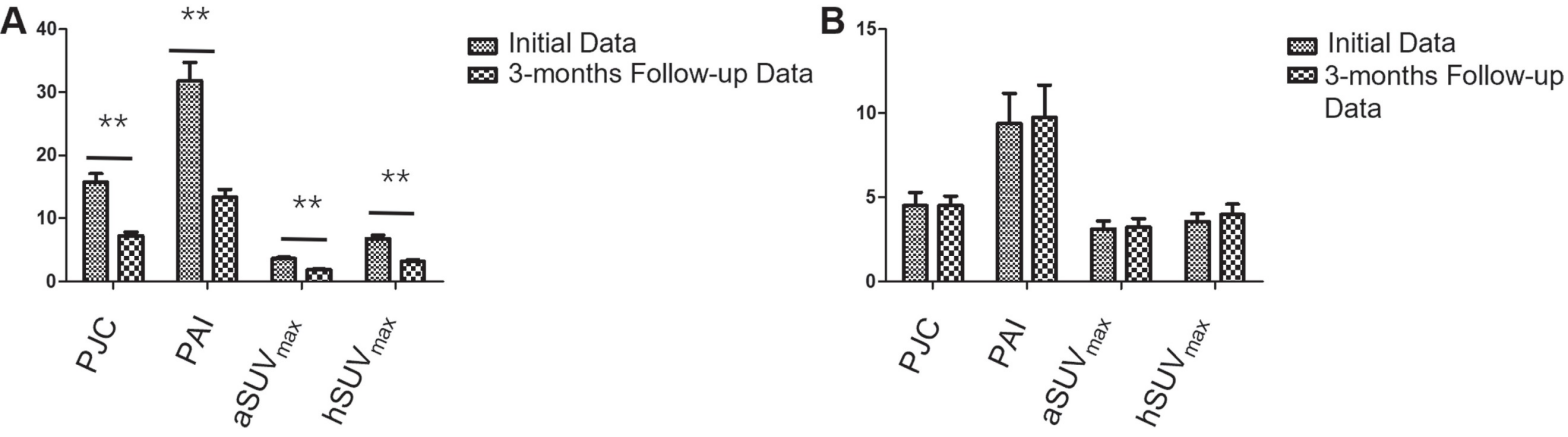
Comparison of initial and follow-up PET parameters in responders and non-responders of participants. Over a 3-month follow-up period, (A) 20 responders showed significant reductions in PJC, PAI, aSUV_max_, and hSUV_max_ values, decreasing from 15.8 ± 5.9, 31.8 ± 13.2, 3.6 ± 1.1, and 6.8 ± 2.3 to 7.3 ± 2.5, 13.4 ± 5.5, 1.9 ± 0.6, and 3.2 ± 1.1 (all *P* < 0.001). (B) Conversely, no significant changes were observed in the 8 non-responders, with values remaining relatively stable from 4.6 ± 2.2, 9.4 ± 5.0, 3.1 ± 1.3, and 3.6 ± 1.3 to 4.4 ± 1.6, 9.8 ± 5.4, 3.2 ± 1.4, and 4.0 ± 1.8 (all *P* > 0.05). ^**^ *P* < 0.01.

**Figure 6:**
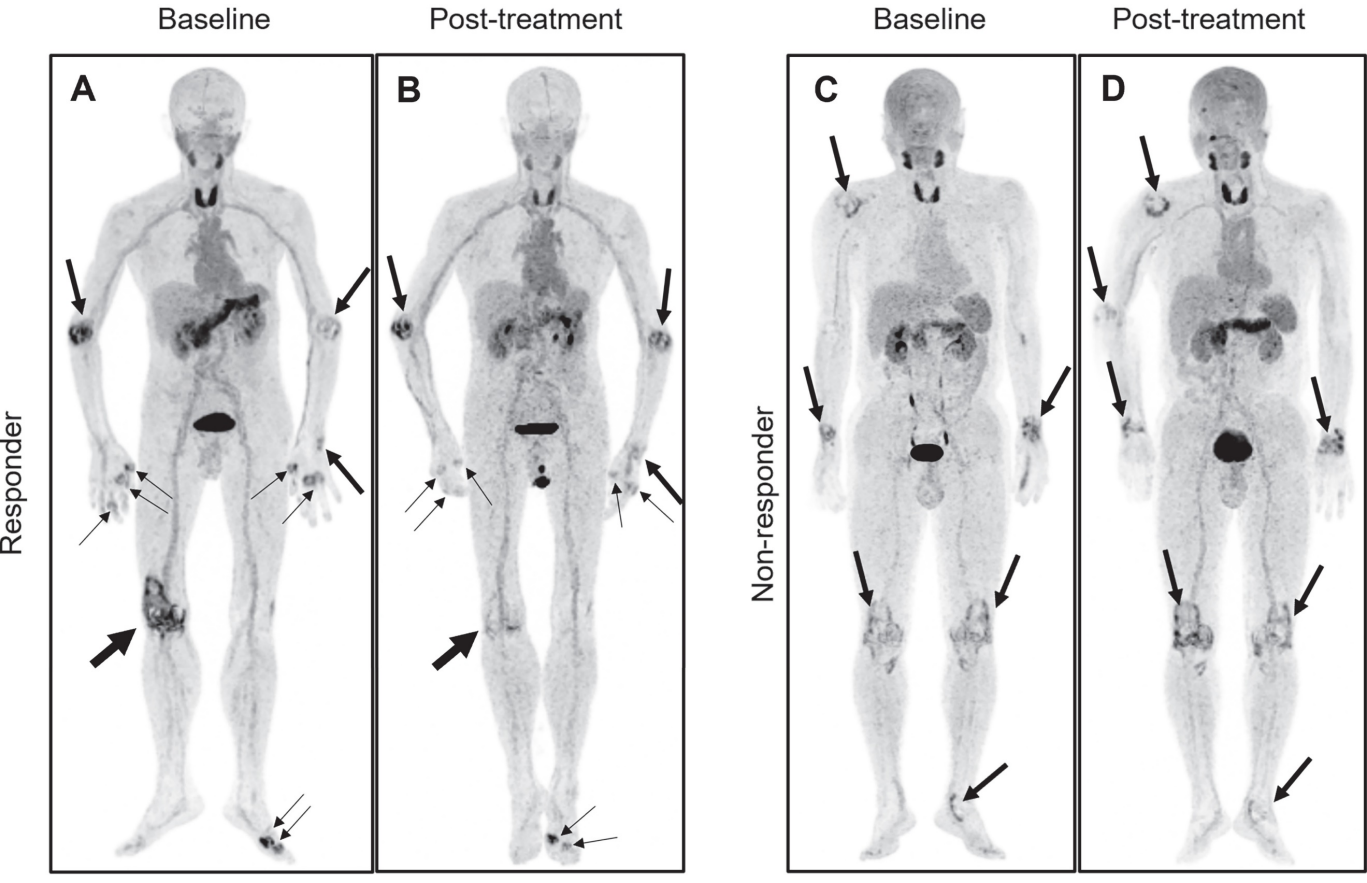
Representative pre- and posttreatment [^18^F]AlF-FAPI-RGD PET/CT images in the 2 participants with different responses undergoing tight control treatment. Baseline and follow-up whole-body MIP images (A and B) from a 71-year-old male participant (No. 3) reveal a significant reduction in FAP-RGD uptake in most affected joints (thick and thin arrows), notably in the right knee. The DAS28-ESR, DAS28-CRP, CDAI, and SDAI scores decrease from 7.4, 6.2, 39.0, and 61.5 to 5.0, 3.5, 16.3, and 18.0, respectively. Similarly, PJC, PAI, aSUV_max_, and hSUV_max_ exhibit reductions from 13, 22, 3.5, and 7.4 to 10, 12, 3.2, and 6.1. Another baseline and follow-up whole-body MIP images (C and D) from a 53-year-old male participant (No. 4) demonstrate an increased FAP-RGD uptake in most of the same joints (thick arrows) compared to the previous scan, while showing a decreased uptake in the left knee and left ankle. The DAS28-ESR, DAS28-CRP, CDAI, and SDAI scores change from 4.3, 3.6, 13.0, and 16.8 to 4.6, 3.8, 13.0, and 21.7, respectively. PJC, PAI, aSUV_max_, and hSUV_max_ change from 7, 14, 1.7, and 1.9 to 6, 15, 1.6, and 2.1.

All 28 individuals had a follow-up PET/CT scan using [^18^F]AlF-FAPI-RGD after the initial evaluation, with a 3-month (90 ± 3 days) gap. Among these, 22 had a decrease in PJC, while 23 saw reductions in PAI, aSUV_max_, and hSUV_max_ post-treatment. The PET measures (PJC, PAI, aSUV_max_, and hSUV_max_) showed a substantial drop post-treatment in the responder cohort (*t* = 5.90, 5.76, 6.46, and 6.20, respectively; all *P* < 0.001), with no notable change in the non-responder group (all *P* > 0.05) (see Figure 5). The PET/CT images of both responder and non-responder participants are illustrated in Figure 6. Furthermore, there was a notable association between the percentage change in certain PET parameters and modifications in specific clinical parameters (refer to Table 3).

## Discussion

Our first investigation in humans focused on using the [^18^F]AlF-labelled dual-targeting tracer, FAPI-RGD heterodimer, for imaging synovial tissue and monitoring treatment in RA participants. In the present study, we revealed that [^18^F]AlF-FAPI-RGD PET/CT imaging provided high-quality images in RA participants, with clear tracer uptake in affected joints compared to surrounding tissues. Additionally, [^18^F]AlF-FAPI-RGD PET/CT imaging effectively detected joint damage in addition to 28 joint regions in RA participants. Changes in PET parameters before and after 3 months of antirheumatic therapy were also found to be significantly correlated with changes in clinical parameters.

In addition to the 28 joints typically assessed for disease activity in RA, [^18^F]AlF-FAPI-RGD PET/CT could sensitively detect inflammation in other large and small joints throughout the body. These patients exhibiting multiple atypical joint involvement lack a family history of spondylarthritis and frequently present with elevated titers of RF and ACPA, suggesting a potential diagnosis of RA. Nonetheless, additional testing is required to rule out nonspecific uptake in joints and tendons resulting from osteoarthritis and tendinopathy. This highlights the advantage of [^18^F]AlF-FAPI-RGD PET/CT in evaluating the entire body in a single examination, providing rheumatologists with an additional imaging modality to comprehensively assess disease activity and prognosis in participants with RA.

Interstitial lung disease (ILD) is a significant complication that can occur in patients with RA and can impact their overall prognosis 32. Early screening, diagnosis, and intervention are essential for improving outcomes in RA-ILD. Clinical manifestations of RA-ILD may not always be obvious, with some patients showing subtle or no symptoms. Rheumatologists usually use high-resolution computed tomography scans to detect ILD, but this method can sometimes lead to cases being overlooked. In this study, we identified a total of 22 cases of pulmonary lesions with diverse manifestations among 28 subjects. All subjects exhibited varying levels of FAPI-RGD uptake, as well as differing titers of RF and ACPA. These findings suggest that some of the 22 patients with RA may be complicated by ILD; however, high-resolution computed tomography and additional examinations are necessary to confirm this conclusion. Notably, the 22 participants primarily presented with joint swelling or pain, rather than significant respiratory symptoms. These results indicate a potential new imaging approach for the early detection of ILD in RA patients who exhibit atypical respiratory symptoms.

Evaluating disease activity and treatment effectiveness at different time points is essential in managing patients with RA. Multiple clinical, biochemical, and imaging techniques can be utilized for this objective, each presenting unique strengths and limitations. Notably, certain modern imaging modalities, such as Doppler ultrasound and contrast-enhanced MRI, demonstrate greater sensitivity in detecting RA compared to traditional clinical evaluations. Ultrasound is capable of detecting inflammation in the synovial membrane and erosive changes in bone during the early stages of RA; nonetheless, it requires skilled operators and can be quite time-intensive 33. On the other hand, MRI is proficient in identifying edema in the bone marrow and inflammation in the soft tissues surrounding the joints; however, it is unsuitable for individuals with metal implants or those who suffer from claustrophobia 34. We found a higher positivity rate of [^18^F]AlF-FAPI-RGD PET/CT in detecting affected joints in participants with RA compared with TJC or SJC through clinical assessment. Furthermore, we observed that 131 out of 415 (31.6%) arthritic joints showed no signs of tenderness or swelling during clinical assessment, yet were identified as affected on PET/CT scans. This discrepancy may be attributed to the potential oversight by rheumatologists in detecting a small number of affected joints during clinical evaluation. However, we propose that the main reason for this discrepancy lies in the [^18^F]AlF-FAPI-RGD PET's ability to detect neovascularization or fibroblast activation in the synovial membrane of joints at an early disease stage, especially in small joints, prior to the manifestation of clinical symptoms in RA participants. Nevertheless, additional validation through larger sample size studies and immunohistochemical analysis is required. Detecting subclinical RA early and promptly providing antirheumatic medication therapy is crucial for preventing joint damage, making these findings valuable insights for rheumatologists.

On the other side, we found of the total 415 joints detected at [^18^F]AlF-FAPI-RGD PET/CT or physical examination, 73 tender or swollen joints (17.6%) showed no uptake of [^18^F]AlF-FAPI-RGD. In addition to the signs and symptoms of joint pain or swelling that may be caused by active rheumatoid disease or mechanical and degenerative changes (secondary osteoarthritis), as mentioned previously in the literature 17, it is suggested that these symptoms could also be attributed to the progression of the disease to the stage of joint fibrosis or ankylosis. At this stage, although the RA patient may still subjectively perceive swelling or tenderness in the joints, there are few angiogenesis and FLSs in the synovial tissue, which may not be sufficient to cause high uptake of FAPI-RGD. Our study revealed that while most quantitative parameters from [^18^F]AlF-FAPI-RGD PET/CT were associated with clinical disease activity measures, there was no notable correlation between PET parameters and van der Heijde mTSS. This reinforces the hypothesis. Fig 2 displays radiographs of the hands and wrists of participant 18, which exhibit slight narrowing and blurring of the interphalangeal joint spaces, along with swelling of the periarticular soft tissues and indistinct carpal joint spaces. Notably, there was no significant increase in FAP-RGD uptake detected at the corresponding sites. This phenomenon may result from the prolonged course of RA, during which lesions in the affected joints can become chronic. Furthermore, the expression of angiogenesis and FLSs in the synovial tissue may not be significantly elevated.

At the 3-month follow-up, a significant correlation was observed between the percentage change in specific PET parameters and the changes in certain clinical parameters. The response group showed a notable reduction in PET parameters after treatment. All 4 [^18^F]AlF-FAPI-RGD PET/CT parameters were deemed useful for evaluating response in RA participants. Our study confirmed similar findings to those of Zhu et al. [22]And Kavanal et al. 23 in ^68^Ga-RGD_2_ PET/CT imaging, by utilizing CDAI or DAS28-ESR and EULAR response criteria. We extended these results to a cohort of RA participants using [^18^F]AlF-FAPI-RGD PET/CT.

The limitations of this study include the small size of the PET/CT follow-up cohort and the short duration of the follow-up period. Although [^18^F]AlF-FAPI-RGD PET/CT could sensitively reflect changes in the activity of RA patients after three months of treatment, these factors restrict the predictive value of PET/CT for long-term outcomes of antirheumatic treatment. Additionally, [^18^F]AlF-FAPI-RGD appears to exhibit physiological uptake in the nail beds; consequently, the evaluation of the distal interphalangeal joints may be compromised. Furthermore, synovial tissue in enrolled participants was not immunohistochemically stained for FAP and α_v_β_3_-integrin to confirm FAPI-RGD uptake specificity. Moreover, the study only utilized [^18^F]AlF-FAPI-RGD PET/CT imaging without head-to-head comparison results of FAPI or RGD PET/CT imaging. Future PET/CT follow-up studies with larger cohorts, including PET/CT examinations with other tracers and corresponding pathological findings, are necessary to validate the efficacy of [^18^F]AlF-FAPI-RGD PET/CT imaging in RA participants.

## Conclusion

The study illustrated that the [^18^F]AlF-labelled dual-targeting tracer FAPI-RGD PET/CT imaging showed heightened uptake in affected joints and lung lesions, serving as a valuable tool for evaluating disease activity and treatment response in RA patients. Additionally, this imaging technique may provide a novel approach for the early detection of subclinical RA and interstitial lung disease present with atypical respiratory symptoms.

## Supplementary Material

Supplementary tables.

## Figures and Tables

**Table 1:** Characteristics in participants (n = 28)

Characteristic	Value
Age (years; mean ± SD)	55 ± 9
Gender	12 males, 16 females
Rheumatoid factor (RF, IU/mL)	566.6 ± 753.3
ACPA (RU/mL)	564.4 ± 310.4
Erythrocyte sedimentation rate (ESR, mm/h)	45.0 ± 27.7
C-reactive protein (CRP, mg/L)	10.8 ± 6.6
Tender joint counts (TJC)^ #^	11.5 ± 7.1
Swollen joint counts (SJC)^ #^	9.0 ± 6.5
VAS of pain	5.9 ± 2.3
PGA of disease activity	6.6 ± 2.7
EGA of disease activity	6.5 ± 2.6
DAS28-ESR^#^	6.4 ± 1.5
DAS28-CRP^#^	5.4 ± 1.3
CDAI^#^	34.0 ± 16.5
SDAI^#^	65.6 ± 47.9
van der Heijde mTSS	32.9 ± 30.8
PJC	13.4 ± 7.1
PAI	25.2 ± 15.5
aSUV_max_	3.5 ± 1.1
hSUV_max_	5.9 ± 2.5

ACPA: anti-citrullinated protein antibodies; VAS: visual analog scale; PGA: patient global assessment; EGA: evaluator global assessment; CDAI: clinical disease activity index; SDAI: simplified disease activity index; DAS28: disease activity score with 28-joint counts; mTSS: modified total Sharp score; PJC: PET joint count; PAI: PET articular index; aSUV_max_: the average SUV_max_ value of the PET-positive joints; hSUV_max_: the highest SUV_max_ value among the PET-positive joints. ^#^ Assessed using 28-joint counts

**Table 2:** Correlation between the uptake of [^18^F]AlF-FAPI-RGD and clinical parameters in participants (n = 28)

Clinical Assessment of Disease Activity	PJC	PAI	aSUV_max_	hSUV_max_
ESR (mm/h)				
*R* value	0.717	0.493	0.424	0.633
*P* value	< 0.001^*^	0.008	0.02	< 0.001^*^
CRP (mg/L)				
*R* value	0.447	0.341	0.363	0.531
*P* value	0.017	0.076	0.049	0.003
SJC				
*R* value	0.500	0.331	0.049	0.258
*P* value	0.007	0.085	0.80	0.17
TJC				
*R* value	0.737	0.637	0.154	0.420
*P* value	< 0.001^*^	< 0.001^*^	0.42	0.026
DAS28-ESR				
*R* value	0.648	0.624	0.030	0.535
*P* value	< 0.001^*^	< 0.001^*^	0.878	0.002
DAS28-CRP				
*R* value	0.636	0.597	0.037	0.568
*P* value	< 0.001^*^	0.001^*^	0.854	0.001^*^
CDAI				
*R* value	0.633	0.615	0.073	0.474
*P* value	< 0.001^*^	< 0.001^*^	0.70	0.008
SDAI				
*R* value	0.140	0.264	0.469	0.686
*P* value	0.476	0.175	0.012	< 0.001^*^
van der Heijde mTSS				
*R* value	-0.153	-0.245	-0.053	-0.052
*P* value	0.438	0.210	0.789	0.793

ESR: erythrocyte sedimentation rate; CRP: C-reactive protein; SJC: swollen joint count; TJC: Tender joint count; DAS28: disease activity score with 28-joint counts; CDAI: clinical disease activity index; SDAI: simplified disease activity index; PJC: PET joint count; PAI: PET articular index; aSUV_max_: the average SUV_max_ value of the PET-positive joints; hSUV_max_: the highest SUV_max_ value among the PET-positive joints; mTSS: modified total Sharp score. ^*^ Bonferroni's correction for multiple correlations: *P* < 0.001.

**Table 3:** Correlation between changes in PET parameters and disease activity before and after 3 months of treatment in participants

Change in Disease Activity	%ΔPJC	%ΔPAI	%ΔaSUV_max_	%ΔhSUV_max_
%ΔDAS28-ESR				
*R* value	0.690	0.851	0.811	0.783
*P* value	0.01	< 0.001^*^	0.001^*^	0.003^*^
%ΔDAS28-CRP				
*R* value	0.641	0.806	0.741	0.713
*P* value	0.03	0.002^*^	0.006	0.009
%ΔCDAI				
*R* value	0.719	0.844	0.795	0.743
*P* value	0.008	0.001^*^	0.002^*^	0.006
%ΔSDAI				
*R* value	0.751	0.865	0.816	0.706
*P* value	0.005	< 0.001^*^	0.001^*^	0.01

%Δ: percentage change; DAS28: disease activity score with 28-joint counts; ESR: erythrocyte sedimentation rate; CRP: C-reactive protein; CDAI: clinical disease activity index; SDAI: simplified disease activity index; PJC: PET joint count; PAI: PET articular index; aSUV_max_: the average SUV_max_ value of the PET-positive joints; hSUV_max_: the highest SUV_max_ value among the PET-positive joints. ^*^ Bonferroni's correction for multiple correlations: *P* < 0.003.

## Abbreviations

ACPA: anti-citrullinated protein antibodies
CDAI: clinical disease activity index
CRP: C-reactive protein
DAS28: disease activity score with 28-joint counts
DMARDs: disease-modifying antirheumatic drugs
EGA: evaluator global assessment
ESR: erythrocyte sedimentation rate
FAP: fibroblast activation protein
FDG: fluoro-2-deoxyglucose
FLSs: fibroblast-like cells
ILD: interstitial lung disease
MRI: magnetic resonance imaging
mTSS: modified total Sharp score
PET/CT: positron emission tomography/computed tomography
PGA: patient global assessment
RA: rheumatoid arthritis
RF: rheumatoid factor
RGD: Arg-Gly-Asp
SDAI: simplified disease activity index
SJC: swollen joint count
SUV_max_: maximum standardized uptake value
TJC: tender joint count
VAS: visual analog scale
