# Supplementary material for: Dual targeting heterodimer PET tracer [18F]AlF‑FAPI‑RGD in patients with rheumatoid arthritis: a pilot exploratory study
Source: Theranostics. 2024 Oct 21;14(18):7042–53. doi: 10.7150/thno.102627 (PMC11610132; doi:10.7150/thno.102627)
Supplement: Supplementary file 1 — Supplementary tables. [file thnov14p7042s1.pdf]

Supplementary Table 1. Characteristics of the individual population (n=28)

No.	Age (years)	Gender	Disease stage	van der Heijde mTSS	RF (IU/mL)	ACPA (RU/mL)	Medication after enrollment
1	52	Male	refractory	6	1410	628	MTX, NSAIDS, ADA
2	51	Female	relapsing	35	988	515.2	LEF, PRED
3	71	Male	new onset	16	308	341	MTX, PRED
4	53	Male	new onset	12	9.19	425	MTX, NSAIDS
5	54	Female	refractory	8	20.40	618.2	MTX, PRED, ADA
6	66	Female	relapsing	52	2730	150.67	PRED, LEF
7	60	Male	new onset	60	971	976	PRED, MTX
8	58	Female	refractory	25	3.25	112	NSAIDS, HCQ
9	55	Female	new onset	16	38.10	517	PRED, MTX, HCQ
10	65	Female	refractory	85	24.7	1654	MTX, NSAIDS, ADA
11	52	Male	refractory	16	654	873	PRED, MTX
12	49	Female	refractory	21	234	756.1	PRED, MTX, HCQ, LEF
13	63	Female	refractory	25	37.7	413	NSAIDS, MTX
14	53	Male	relapsing	14	2130	935	PRED, MTX, HCQ
15	50	Female	relapsing	116	558	548	MTX, TOC, NSAIDS
16	45	Male	new onset	14	69.01	676	PRED, MTX
17	59	Male	refractory	6	118	320.73	PRED, MTX
18	69	Female	relapsing	128	474	435	MTX, HCQ, NSAIDS
19	35	Male	refractory	15	135	386	PRED, MTX
20	57	Female	new onset	28	76	547	NSAIDS, MTX
21	62	Female	refractory	22	5.37	231.4	PRED, HCQ
22	65	Male	refractory	13	117.2	427.3	MTX, PRED, ADA
23	50	Female	refractory	36	281	575	NSAIDS, MTX, HCQ, LEF
24	43	Male	relapsing	42	762.1	563.2	LEF, PRED
25	38	Female	refractory	28	40.5	561.2	PRED, MTX
26	52	Female	relapsing	16	1867	923	PRED, MTX, HCQ

27	60	Male	new onset	20	45	453.2	NSAIDS, MTX, HCQ
28	47	Female	relapsing	45	1757.1	243	NSAIDS, LEF, PRED

No.	Joint involvement	CRP (mg/L)	ESR (mm/h)	Duration of symptoms (month)	Total score of categories
1	6 large joints and 7 small joints	11	61	7	9
2	5 large joints and 9 small joints	19.32	78	9	9
3	3 large joints and 8 small joints	22.46	112	11	9
4	6 large joints and 2 small joints	48.32	108	25	6
5	2 large joints and 2 small joints	0.46	25	37	7
6	4 large joints and 5 small joints	1.12	31	42	8
7	5 large joints and 2 small joints	203	120	48	7
8	3 large joints and 8 small joints	3.82	24	65	7
9	4 large joints and 8 small joints	7.91	84	66	9
10	3 large joints and 12 small joints	7.29	54	120	10
11	5 large joints and 13 small joints	69.81	83	1	8
12	6 large joints and 15 small joints	75.20	120	12	9
13	5 large joints and 8 small joints	10.65	84	12	9
14	6 large joints and 18 small joints	6.45	76	12	10
15	6 large joints and 5 small joints	63.5	120	122	8
16	3 large joints and 4 small joints	106.29	120	123	7
17	4 large joints and 8 small joints	14.24	40	123	8
18	3 large joints and 4 small joints	22.1	49	125	7
19	2 large joints and 9 small joints	14.24	40	13	8
20	3 large joints and 5 small joints	106.29	120	13	7
21	2 large joints and 9 small joints	3.82	24	2	7
22	2 large joints and 4 small joints	0.46	23	370	7
23	5 large joints and 16 small joints	75.20	120	38	9
24	3 large joints and 9 small joints	19.32	78	49	9

25	4 large joints and 9 small joints	10.65	84	5	8
26	5 large joints and 20 small joints	6.45	76	6	10
27	3 large joints and 9 small joints	7.91	84	62	9
28	4 large joints and 8 small joints	1.12	31	65	10

mTSS: modified total Sharp score; RF: rheumatoid factor; ACPA: anti-citrullinated protein antibodies; MTX: methotrexate; LEF: leflunomide; PRED: prednisone; NSAIDS: nonsteroidal anti-inflammatory drugs; HCQ: hydroxychloroquine; TOC: tocilizumab; ADA: adalimumab.

Supplementary Table 2. The comparison of PET parameters between RA patients with new-onset active disease and those with relapsing or continuous active disease (n = 28)

Parameter	New-onset active disease (n = 7)	Relapsing or continuous active disease (n = 21)	*t*	*P* value
PJC	11.7 ± 2.9	12.8 ± 8.3	-0.518	0.609
PAI	30.4 ± 10.7	23.7 ± 16.5	1.001	0.326
$aSUV_{max}$	4.3 ± 0.6	3.2 ± 1.2	2.357	0.026*
$hSUV_{max}$	7.2 ± 2.6	5.4 ± 2.4	1.599	0.122

PJC: PET joint count; PAI: PET articular index; $aSUV_{max}$: the average SUV_{max} value of the PET-positive joints; $hSUV_{max}$: the highest SUV_{max} value among the PET-positive joints.

* $P < 0.05$.

Supplementary Table 3. The detection results of [^{18}F]AlF-FAPI-RGD PET/CT in other positive joints apart from the 28 joint regions and lesions in non-articular tissues

No.	Other positive joints	Interstitial lung disease	Additional findings
1	MTP and LIP	Nodules and plaques	PMSA, TSST
2	ATA, MTP and LIP	None	PMSA, TSST
3	MTP	None	None
4	ATA, ankle and MTP	Plaques and streaks	None
5	Ankle	Streaks and ground-glass opacities	Rib fracture
6	Ankle, MTP and LIP	Nodules and streaks	None
7	TPM, ATA, SNC, hip, ankle and MTP	Nodules, plaques and streaks	PMSA, TSST
8	SNC	Plaques	None
9	DIP, ankle, MTP and LIP	Nodules and streaks	None
10	Ankle, MTP and LIP	Ground-glass opacities	None
11	TPM, ATA, SNC, hip, ankle, MTP and LIP	None	PMSA, TSST
12	Hip, ankle, MTP and LIP	Nodules	None
13	DIP, ankle and LIP	Plaques	None
14	ATA, SNC, ankle, MTP and LIP	Plaques, streaks and ground-glass opacities	None
15	ATA, ankle and LIP	None	None
16	TPM, ankle and LIP	Nodules	TSST
17	Ankle and LIP	Nodules, streaks and ground-glass opacities	None
18	Ankle and LIP	None	None
19	Ankle and LIP	Nodules, streaks and ground-glass opacities	None
20	TPM, ankle and LIP	Nodules and streaks	None
21	SNC	Plaques	None
22	Ankle	Streaks and ground-glass opacities	None
23	Hip, ankle, MTP and LIP	Nodules	None
24	ATA, MTP and LIP	None	PMSA, TSST
25	DIP, ankle and LIP	Plaques	None
26	ATA, SNC, ankle, MTP and LIP	Plaques, streaks and ground-glass opacities	None
27	DIP, ankle, MTP and LIP	Nodules and streaks	None
28	Ankle, MTP and LIP	Nodules and streaks	None

MTP: metatarsophalangeal; LIP: interphalangeal joint of the lower extremities; ATA: atlantoaxial; TPM: temporomandibular; SNC: sternoclavicular; DIP: distal interphalangeal; PMSA: paraspinal muscles of the subscapularis angle; TSST: tissues surrounding the sciatic tuberosity.
